# Mitochondrial genome of the freshwater annelid *Manayunkia occidentalis* (Sabellida: Fabriciidae)

**DOI:** 10.1080/23802359.2020.1815604

**Published:** 2020-09-03

**Authors:** Ekin Tilic, Stephen D. Atkinson, Greg W. Rouse

**Affiliations:** a Institute of Evolutionary Biology, University of Bonn, Bonn, Germany; b Scripps Institution of Oceanography, University of California, San Diego, CA, USA; c Department of Microbiology, Oregon State University, Corvallis, OR, USA

**Keywords:** Annelida, mitogenome, polychaete

## Abstract

Here, we report the 15,103 bp mitochondrial genome of the freshwater fabriciid tubeworm *Manayunkia occidentalis*. We recovered 13 protein-coding genes, 2 rRNA, and 22 tRNA. The gene order is consistent with the conserved pattern observed in most annelids.

*Manayunkia occidentalis* (Atkinson et al. 2020) is a freshwater fabriciid that has an important role in the complex life cycles of commercially important, salmonid-infecting, myxozoan parasites (Bartholomew et al. 2006; Atkinson et al. 2020). Also, *M. occidentalis* is one of the few freshwater annelid species (outside Clitellata), and forms a clade with the marine-dwelling *Echinofabricia*, which is sister to the other Fabriciidae (Huang et al. 2011). Fabriciidae is one of three taxa within Sabellida, and is sister to Sabellidae (feather-duster worms) and Serpulidae (calcareous tube worms) (Tilic et al. 2020). As next-gen sequencing methods become more common, the number of available mitochondrial genomes for annelids is increasing rapidly. However, complete mitochondrial genome sequences are not necessarily useful to resolve deep nodes of annelid phylogeny (Figure 1) (Weigert et al. 2016), but they can reveal in-group relationships among annelid clades at various levels of inclusiveness (Li et al. 2015; Zhang et al. 2018). Furthermore, the arrangement of protein-coding mitochondrial genes and tRNAs is an additional dataset that can help identify conserved and novel gene arrangements in deep phylogenetic lineages (Weigert et al. 2016). Of the 77 available annelid mitogenomes, 33 are from Clitellata, 13 from Siboglinidae, and 18 from Aciculata. There are no complete mitochondrial genomes available for the vast majority of annelid taxa. Herein we present the first mitochondrial genome for Fabriciidae. The only other mitochondrial genome available for Sabellida is that of the Christmas tree worm *Spirobranchus giganteus* (Pallas 1766) (Serpulidae).

**Figure 1.**
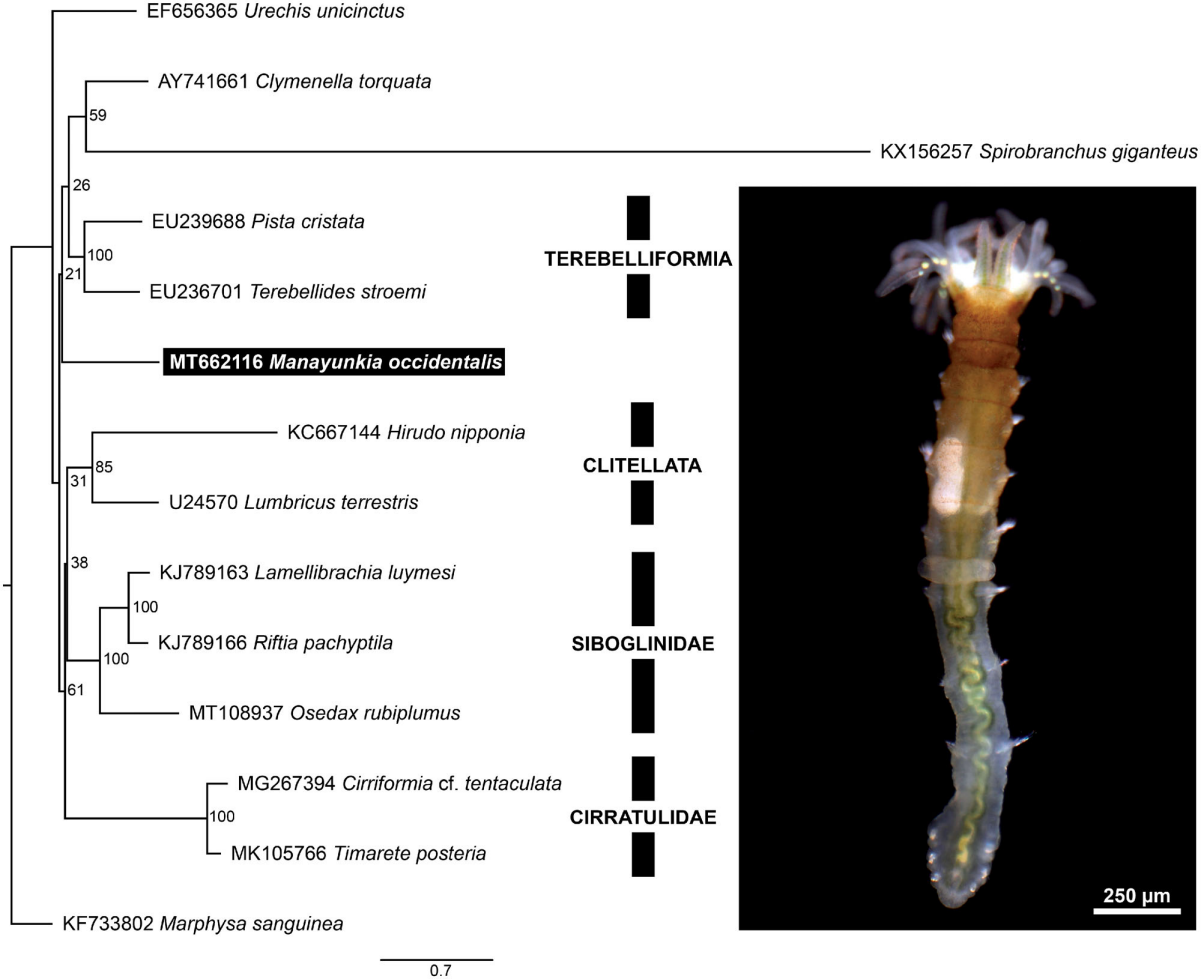
Maximum likelihood (ML) tree based on the concatenated nucleotide sequences of 13 mitochondrial protein-coding genes. Bootstrap support values are indicated at each node. *Marphysa sanguinea* (KF733802) was chosen as an outgroup.

Specimens were obtained from a laboratory culture of annelids, sourced from the Klamath River, California (41°52′02.6″N 122°48′37.4″W). Three individuals were harvested and frozen at −80° C until their DNA was extracted using a DNeasy Blood and Tissue kit (Qiagen Inc., Valencia, CA) according to the manufacturer’s protocol. Due to low total DNA recovered, DNA from the three annelids was pooled, and a library was prepared using a Wafergen PrepX Total Genomic DNA kit (Takara Bio USA, Mountain View, CA). DNA was sequenced with 150 bp paired-end reads on an Illumina HiSeq3000 platform (Illumina, San Diego, CA) at Oregon State University’s Center for Genome Research and Biocomputing.

The complete circular mitochondrial genome of *M. occidentalis* was de novo assembled with Novoplasty v.4.0 (Dierckxsens et al. 2017) and MitoFinder v.1.3 (Allio et al. 2020) from a randomly sampled subset of total genomic reads (1,905,901 paired end). Both methods recovered one identical, circular mtDNA contig of the entire mitochondrial genome. For Novoplasty, the published COI sequence of *M. occidentalis* (MN991228) was used as a seed sequence. The average coverage of the assembly was 82.62×. The mitochondrial genome was annotated using the annotation pipeline integrated into MitoFinder.

Nucleotide sequences of protein-coding genes of 12 annelids belonging to Sedentaria were chosen from available mitochondrial genomes, together with *M. occidentalis* and an outgroup annelid, *Marphysa sanguinea* (Montagu 1813). These were aligned with MAFFT (Katoh and Standley 2013), and a maximum-likelihood analysis of the concatenated supermatrix was conducted with IQTree (Nguyen et al. 2015). Substitution models and partitions were determined automatically.

The mitochondrial genome of *M. occidentalis* (GenBank Accession MT662116) is 15,103 bp long. The average length of the other 77 available annelid mitochondrial genomes is 15,404 bp; with *Spirobranchus giganteus* having the longest (22,058 bp) and *Erpobdella octoculata* the shortest (14,407 bp). The GC content of the *M. occidentalis* mitochondrial genome is 28.33%.

We identified 13 protein coding genes, 2 rRNAs and 22 tRNAs. The mitochondrial gene order in *M. occidentalis* is consistent with the pattern observed in most annelids (Weigert et al. 2016). Interestingly, the phylogenetically closest mitogenome to *M. occidentalis* is that of *Spirobranchus giganteus*, which has a unique gene order and is divergent from other annelids (Seixas et al. 2017). This explains the placement and long branch of *S. giganteus* in Figure 1, and in it not being recovered as sister group to *M. occidentalis*. As more annelid mitogenomes become available, more exceptions to the conserved gene order are being revealed: another example is the small meiofaunal annelid *Dimorphilus gyrociliatus* (Schmidt 1857), which has a different gene order and lacks trnS1 (David and Halanych 2017). To better understand the evolution of mitochondrial gene order in Annelida, more complete mitochondrial genomes spanning the group’s phylogenetic diversity, and representing diverse taxa need to be sequenced.

## Data Availability

All sequence data included and generated in the study are deposited to NCBI GenBank (https://www.ncbi.nlm.nih.gov) and their accession numbers are listed in Figure 1.
